# CPSF30 at the Interface of Alternative Polyadenylation and Cellular Signaling in Plants

**DOI:** 10.3390/biom5021151

**Published:** 2015-06-08

**Authors:** Manohar Chakrabarti, Arthur G. Hunt

**Affiliations:** Department of Plant and Soil Sciences, University of Kentucky, Lexington, KY 40546-0312, USA; E-Mail: mchak2@uky.edu

**Keywords:** mRNA 3' end formation, alternative polyadenylation, cleavage and polyadenylation specificity factor (CPSF), CPSF30, stress response

## Abstract

Post-transcriptional processing, involving cleavage of precursor messenger RNA (pre mRNA), and further incorporation of poly(A) tail to the 3' end is a key step in the expression of genetic information. Alternative polyadenylation (APA) serves as an important check point for the regulation of gene expression. Recent studies have shown widespread prevalence of APA in diverse systems. A considerable amount of research has been done in characterizing different subunits of so-called Cleavage and Polyadenylation Specificity Factor (CPSF). In plants, CPSF30, an ortholog of the 30 kD subunit of mammalian CPSF is a key polyadenylation factor. CPSF30 in the model plant *Arabidopsis thaliana* was reported to possess unique biochemical properties. It was also demonstrated that poly(A) site choice in a vast majority of genes in *Arabidopsis* are CPSF30 dependent, suggesting a pivotal role of this gene in APA and subsequent regulation of gene expression. There are also indications of this gene being involved in oxidative stress and defense responses and in cellular signaling, suggesting a role of CPSF30 in connecting physiological processes and APA. This review will summarize the biochemical features of CPSF30, its role in regulating APA, and possible links with cellular signaling and stress response modules.

## 1. Introduction

**Figure 1:**
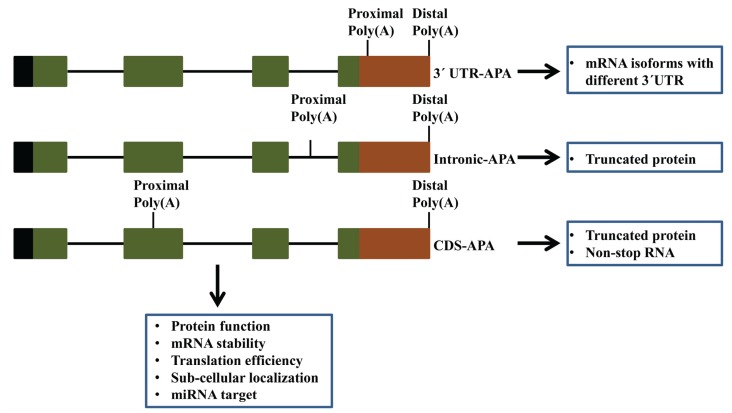
Different types of alternative polyadenylation and their possible consequences on gene expression. A hypothetical gene with four exons is used for schematic representation of different kinds of APA. Exons, 5' UTRs and 3' UTRs are represented with green, black and brown boxes, respectively, while introns are depicted with black horizontal lines. Proximal and distal poly(A) sites are shown with black vertical lines.

The formation of the mature 3' end of mRNA in the nucleus is a key step in gene expression in all eukaryotes. This process involves identification of characteristic polyadenylation (poly(A)) signals in the pre-mRNAs by a multiprotein complex, followed by cleavage of pre-mRNAs and subsequent polyadenylation at the nascent 3' hydroxyl group of the processed transcripts [1,2,3,4]. The polyadenylation process is intrinsically associated with transcription initiation, elongation, termination and mRNA translocation from nucleus to cytosol [5,6,7,8]. Research in diverse eukaryotic organisms has brought forth several examples of widespread occurrences of multiple poly(A) sites in genes and differential usage of multiple poly(A) sites, leading to a phenomenon termed as alternative polyadenylation (APA). A schematic representation of different types of APA is depicted in Figure 1. As shown (“Intronic APA” and “CDS APA” in Figure 1), APA can lead to the production of different transcripts with different protein-coding potential and subsequently distinct proteins [3,9,10,11,12,13,14]. By remodeling 3'-UTRs (“3'-UTR APA” in Figure 1) and altering the regulatory potential of these regions (through inclusion or exclusion of microRNA target sites or motifs that bind proteins that mediate different processes), APA can regulate gene expression by altering transcript stability, translatability and subcellular localization [15,16,17,18,19,20,21,22]. The prevalence of APA is widespread and has been suggested as having roles during growth and development [21,23,24]. For example, global shortening of 3' UTR through APA has been observed in cancer cells, indicating a potential role of APA in oncogene activation [25]. APA has also been implicated in several other human diseases including thalassemia, thrombophilia, oculopharyngeal muscular dystrophy, neurodegenerative diseases, IPEX syndrome and anxiety to name several [17,26,27,28,29,30,31]. Recently, APA was reported to be involved in self-renewal of embryonic stem cells and re-programming of somatic cells, suggesting a role for APA in developmental processes [32].

Perhaps the most well studied example in plants depicting the role of APA in developmental processes is the regulation of flowering time. Numerous core and accessory subunits of the polyadenylation complex have been linked with this process. This includes FY, an ortholog of yeast Pfs2p and a core subunit of plant cleavage and polyadenylation specificity factor (CPSF) [33]. FY in turn acts with another RNA binding protein FCA to facilitate intronic polyadenylation of *FCA*-encoded transcripts. Another RNA binding protein (FPA) along with two core polyadenylation factor subunits CstF64 and CstF77 regulates APA of antisense transcripts encoded by the flowering time regulator gene FLC, and these antisense transcripts are involved in the regulation of *FLC* sense transcript expression through chromatin modification [33,34,35]. A genome wide study with the *Arabidopsis fpa* mutant revealed a more general role for FPA in mRNA 3' end formation and transcription termination, raising the possibility of the involvement of other RNA binding proteins in the mRNA 3' end formation [36]. Core subunits of plant CPSF complex, CPSF73 (I) and CPSF73 (II), also have roles in flower development in *Arabidopsis* [37,38].

In terms of the generation of different mRNA isoforms, beyond the numerous examples that relate to flowering time, APA has been associated with other growth and developmental processes in plants. These instances include the cases of S locus mediated self-incompatibility in *Brassica* and amino acid lysine catabolism [39,40,41]. Evidence for APA has also been documented for genes encoding ICE (Inducer of CBF Expression) transcription factors in grapes, which are implicated in cold acclimation [42].

With the advancement in next generation sequencing technology, plant polyadenylation research has benefitted immensely. Consequently, it has been determined that APA affects more than 70% of genes in different plants [12,43,44,45]. Subsequently, research on the regulation of poly(A) site choice, the roles of different poly(A) factors in this regard, and the physiological and developmental relevance of APA has taken on new significance.

The mammalian and yeast polyadenylation complexes can be resolved into several distinct biochemical sub-complexes. In mammals, one such sub-complex is the so-called Cleavage and Polyadenylation Specificity Factor (CPSF). CPSF is comprised of six subunits, CPSF160, CPSF100, CPSF73, CPSF30, Fip1 and Wdr33 [46,47,48]. These subunits are widely conserved in eukaryotes, including plants [48]. Of these six subunits, CPSF30 (Cleavage and Polyadenylation Specificity Factor 30 k D subunit) warrants special attention because of its unique biochemical and molecular attributes and possible links to cellular signaling systems. In this context, we will summarize recent research on plant CPSF30 orthologs that provides insight into mechanisms of APA, of the physiological consequences of APA in plants, and of the means by which cellular signaling pathways impact APA.

## 2. CPSF30 in Plant is a Key Polyadenylation Factor with Unique Biochemical Features

Mammals and yeast possess a distinctive zinc finger protein (CPSF30 and Yth1p, respectively) that is a core subunit of the polyadenylation complex. This protein is relatively small and possesses, among other features, a distinctive and evolutionarily-conserved array of five CCCH-type zinc finger motifs. In mammals, this protein is a target of influenza virus-encoded NS1 proteins, with the NS1-CPSF30 interaction being one mechanism for the shut-down of host cell gene expression [49]. Additionally, in mammals, CPSF30 seems to associate with the pre-mRNA at or near the AAUAAA polyadenylation signal; this CPSF30-RNA interaction was found to be critical for the interaction between CPSF complex and the RNA, suggesting a vital role of CPSF30-RNA interaction in the mRNA 3' processing [50].

**Figure 2:**
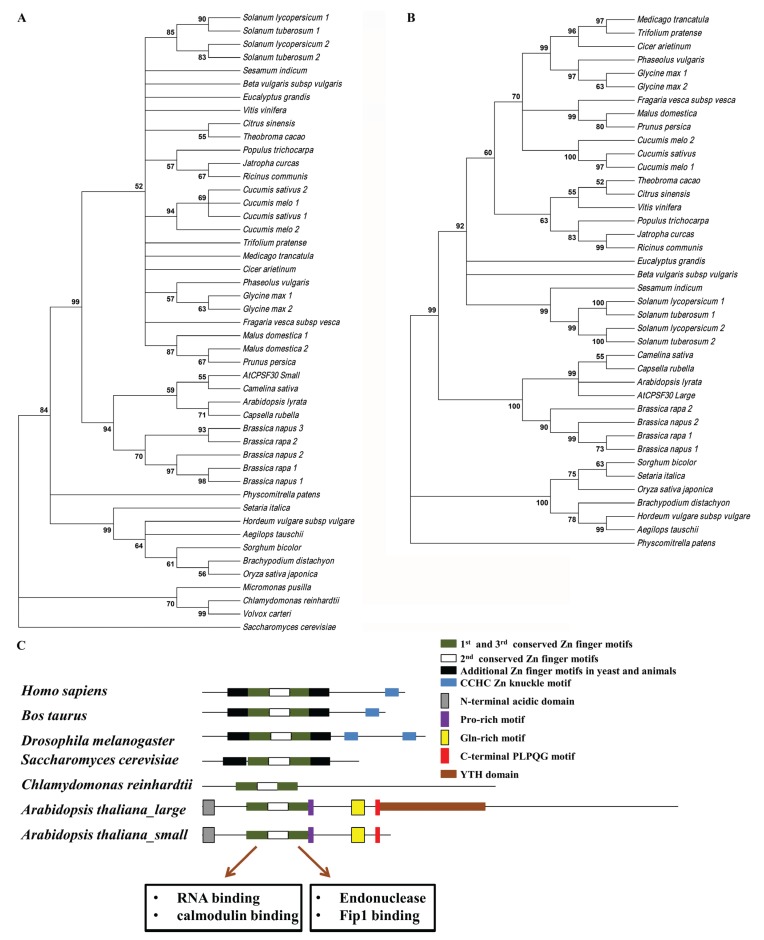
Amino acid sequence alignment and domain architecture analysis of CPSF30. (**A**) Tree showing the results of alignments of the small CPSF30 polypeptide (corresponding to the 28 kD polypeptide encoded by the *Arabidopsis* At1g30460 locus) from diverse plant species, algae, and yeast. (**B**) Tree showing the results of alignments of the large CPSF30 polypeptides from diverse plant species (analogous polypeptides are not present in other eukaryotes). Amino acid sequences were obtained from databases as described [48], aligned with ClustalW, and trees were constructed using neighbor-joining statistical method in MEGA6 software [59]. (**C**) Comparison of domain architecture of CPSF30 in different organisms. Three conserved CCCH zinc finger motifs are depicted in green and white horizontal boxes. Additional CCCH zinc finger and CCHC zinc knuckle motifs in other organisms are shown in black and blue horizontal boxes, respectively. Plant specific N-terminal acidic domains, Pro-rich motifs, Gln-rich motifs, C-terminal PLPQG motifs and YTH domains are represented with gray, violet, yellow, red vertical boxes and brown horizontal boxes, respectively.

Plants possess a related polyadenylation complex subunit; however, while the core of the protein (a central array of three zinc finger motifs) is conserved between animals, yeast, and plants, other features of the protein, and of the genes encoding the proteins in different organisms, are very different. Unlike mammals, yeast and *Drosophila*, the gene encoding CPSF30 in plants (in *Arabidopsis thaliana*, this is encoded by *At1g30460*) gives rise to two transcripts generated through the usage of alternative poly(A) sites, and subsequently produces two distinct polypeptides [48,51]. In *Arabidopsis*, the smaller transcript encodes a 28 kD protein, probably analogous to other eukaryotic CPSF30 proteins. The larger transcript encodes a 65 kD polypeptide in which almost all of the smaller, 28 kD polypeptide is fused with a YTH domain-containing polypeptide [51]. Interestingly, other YTH domain family proteins have recently been shown to bind to N^6^-methyladenosine (m^6^A)-modified mRNAs and regulate their stability [52]. Higher plant CPSF30 orthologs are quite diverged from the algae and yeast orthologs (Figure 2A). Moreover, monocots and dicots CPSF30 isoforms fall into distinct clades that reflect the evolution of these different groups of plant species (Figure 2). The smaller and larger of the higher plant CPSF30 isoforms fall into similar clades (Figure 2A,B), suggesting a common ancestor of the complex plant CPSF30 gene. While the sequence alignments are consistent with a single ancestral CPSF30 gene in higher plants, some plant species, such as soybean, tomato, and potato, possesses multiple genes capable of encoding CPSF30, reflecting the evolutionary history of recent large genomic duplication events in these species (Figure 2, Appendix Table A1, and [48]).

Similar to their animal and yeast counterparts, plant CPSF30 proteins possess distinctive arrays of so-called CCCH zinc finger motifs. However, as opposed to the five CCCH type zinc finger motifs seen in animal and yeast CPSF30 proteins, the plant CPSF30 harbors three such motifs. Specifically, plant CPSF30 proteins lack the first and fifth zinc finger motifs and also C-terminal CCHC type zinc knuckle motifs present in *Drosophila*, *Caenorhabditis elegans*, zebrafish and other animals [51,53]. The three zinc finger motifs in the *Arabidopsis* protein (AtCPSF30) have different biochemical functions. The first and third zinc finger motifs are involved in RNA binding and endonuclease activity, respectively, whereas the function of the second zinc finger motif is not precisely known [51,54]. Interestingly, the *Drosophila* ortholog of CPSF30 also possesses endoribonuclease activity [53,55], and the motif that corresponds to the RNA-binding and Fip1-interacting zinc fingers in the AtCPSF30 (the second CCCH motif on the animal and yeast proteins) are also responsible for the corresponding activities of its yeast counterpart [56]. Remarkably, AtCPSF30 possesses a disulfide linkage formed between the side chains of two cysteine residues in the third (endonucleolytic) zinc finger domain, and reduction of this disulfide bond results in loss of endonuclease activity [57,58]. Analogous features have not been reported for animal or yeast CPSF30 proteins, probably because they have not been searched for. Along with three conserved zinc finger domains, CPSF30 in plants possess unique plant-specific motifs, which include an N-terminal acidic domain, a Pro-rich motif downstream of third Zinc finger domain, a C-terminal Gln-rich region, and a conserved PLPQG motif near the C-terminus [51]. A comparison of domain architecture of CPSF30 in different organisms is represented in Figure 2C.

The RNA binding property of AtCPSF30 shows strong cooperativity [51,54], indicating a possibility of self-association. Indeed, it was found that AtCPSF30 can interact with itself [51]. However, the significance of this self-association in AtCPSF30 with regard to its function in the polyadenylation process is not clear. AtCPSF30 also interacts with other polyadenylation factors, including FIPS5 (the *Arabidopsis* ortholog of the yeast Fip1p protein), CPSF100, and CstF77, and may be pictured as a central hub in the protein-protein interaction network of plant polyadenylation complex subunits [38,54,60,61,62]. When expressed transiently in leaf cells, AtCPSF30 localizes in the cytoplasm, but when co-expressed with *Arabidopsis* orthologs of CPSF160 and CPSF73, it localizes in the nucleus, suggesting that interactions with other polyadenylation factors are needed for nuclear localization of AtCPSF30 [63].

## 3. CPSF30, Cellular Signaling, and Plant Growth and Development

In mammals, the influenza virus-encoded protein NS1 interacts with CPSF30, and binding of CPSF30 to NS1 results in repression of polyadenylation [49]. The interaction between CPSF30 and NS1 involves the second and third of the five zinc finger motifs of the mammalian CPSF30 [64], corresponding to the first and second motifs of the plant protein. CPSF30 is expressed at higher levels in lung adenocarcinoma cell lines and tumor tissues as compared to the normal tissues; interestingly, the higher expression of CPSF30-encoding transcripts significantly correlates with the poor survival of the patients [65]. In contrast, knocking-down the expression of CPSF30 with siRNA results in suppression of proliferation of lung cancer cells. Over-expression of CPSF30 in lung cancer cells results in activation of human telomerase reverse transcriptase (hTERT), which was shown to be associated with the proliferation of cancer cells [65,66]. These findings suggest that altering the activity of CPSF30 in different cellular settings may lead to large scale reprogramming of gene expression, and show that interactions with other regulatory factors (specifically, NS1) can alter the activity and function of CPSF30 *in vivo*.

**Figure 3:**
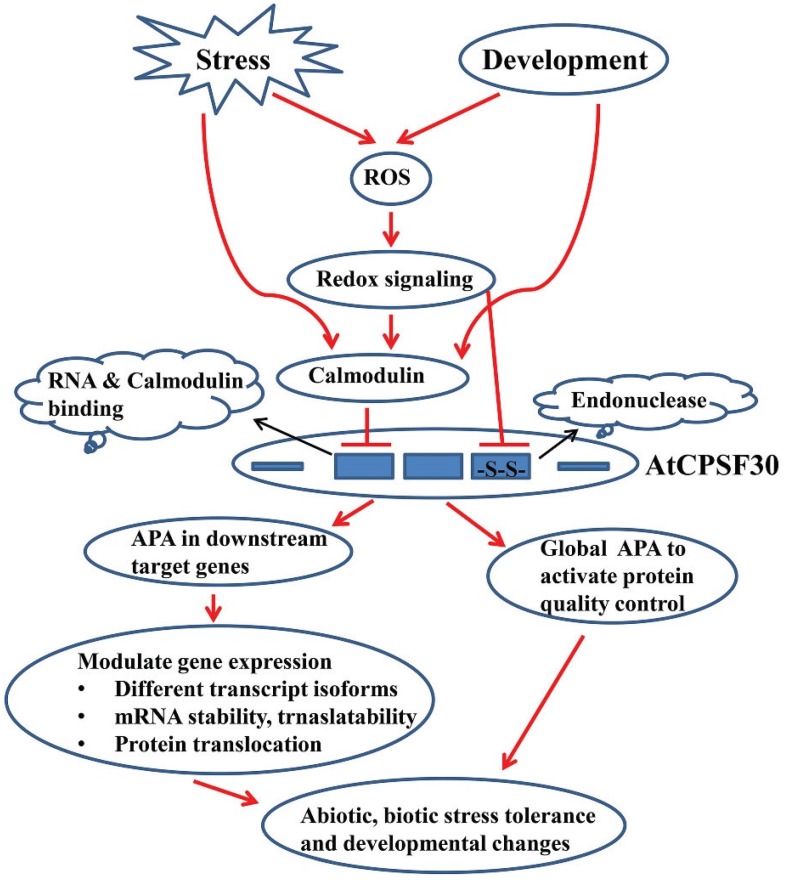
Model depicting possible links between environmental stresses, developmental cues, cellular signaling and AtCPSF30-regulated polyadenylation. Three conserved zinc finger domains of AtCPSF30 are shown with thick blue rectangles. Pertinent biochemical activities are represented as clouds and linked to the respective zinc finger motifs with black arrows. Stress and developmental cues can initiate cellular signaling transmitted through redox and/or calcium-calmodulin mediated signaling cascade, altering RNA binding and/or endonuclease activities of AtCPSF30. This leads to global changes in poly(A) site choice, resulting in numerous biochemical and molecular consequences (induction of protein quality control system, as well as alteration of mRNA stability, translatability and localization). These consequences contribute to the eventual physiological response.

The AtCPSF30 is able to bind calmodulin, and the calmodulin binding domain is juxtaposed with the first zinc finger domain of the protein [51]. Interestingly, this motif corresponds to one of the two motifs involved in the interaction of NS1 with CPSF30 in animals. Calmodulin inhibits RNA binding activity of the AtCPSF30 in a calcium-dependent manner [51], suggesting a possible regulatory role for calmodulin in RNA processing. In plants, calcium acts as a secondary messenger in cellular signaling cascades that perceive environmental and developmental cues, and binding of calcium with various calcium sensors including calmodulin leads to conformational changes, which further modulate its interaction with the downstream targets [67]. Under this backdrop, it is plausible to hypothesize that calcium-dependent calmodulin binding to AtCPSF30 and the concomitant inhibition of RNA binding may provide a link between polyadenylation and responses of plants to environmental stress and developmental processes (Figure 3).

A recent study has tested the functional significance of calmodulin binding activity of AtCPSF30 in diverse developmental processes and in responses to various stimuli [68]. This study reported several novel phenotypes in an *Arabidopsis* mutant (termed *oxt6*) bearing a T-DNA insertion within the first exon of the *At1g30460* locus; this mutant is a null, and produces neither the small AtCPSF30 polypeptide nor the larger CPSF30-YTH protein also encoded by this locus [51]. These phenotypes include lower fertility, reduced lateral root formation, altered responses to plant growth regulators, and modified sensitivity to oxidative stress. Interestingly, transgenes that encode the wild type AtCPSF30 (only the smaller protein encoded by *At1g30460*) can restore wild type growth, development, and responses to the various growth regulators and to oxidative stress. This indicates that, for the battery of phenotypes documented, the larger of the two *At1g30460-*encoded polypeptides seems to be dispensable. In contrast, a transgene encoding a mutant AtCPSF30 isoform that is unable to bind calmodulin (but that retains the other biochemical properties of the protein [51]) only partially restores wild type phenotypes to the *oxt6* mutant. Specifically, this transgene was able to restore wild-type fertility or responses to ethylene or oxidative stress. However, this transgene was unable to restore wild-type lateral root development and responses to other plant hormones, including IAA (indole-3-acetic acid), GA3 (gibberellic acid) and 6-BA (6-benzylaminopurine). These findings suggest that the regulation of different developmental processes and stress responses by AtCPSF30 can be either calmodulin binding-dependent or -independent, raising the possibility that other cellular signaling mechanisms may contribute to AtCPSF30-mediated regulation of developmental and environmental responses.

A recent transcriptomics-based screen for regulators of programmed cell death (PCD) identified the *oxt6* mutant as a strain with a global gene expression profile that “opposed” that of another PCD-related *Arabidopsis* mutant [69]. It was shown that AtCPSF30 is critical for the PCD and additionally that AtCPSF30 is required for resistance to *Pseudomonas syringae*, by modulating both basal resistance and R gene-mediated defense responses [69]. PCD is well recognized for its role in hypersensitive responses in the plant-pathogen interaction, where an unfavorable interaction leads to localized death of host cells around the infection area to limit further progression of the pathogen [70]. This linkage between the AtCPSF30, PCD, and innate immunity furnishes an evidence of role of APA in plant stress response.

It has been well documented that a characteristic response to environmental stresses in plants is the alteration of reactive oxygen species (ROS) at the cellular level. In addition, ROS signaling is known to be involved in developmental processes. Alteration in ROS levels leads to changes in the gene expression, particularly in the genes encoding ROS scavenging enzymes [71,72,73,74]. Increases in ROS levels in turn result in rapid increment of intracellular calcium concentrations, which initiates a cellular signaling cascade [75,76,77,78]. Such calcium bursts at the inception of a stress response may conceivably trigger a calcium-calmodulin dependent regulation of CPSF30 activity; this possibility represents a conceptual link between stress responses and polyadenylation in plants.

As mentioned in the preceding section, AtCPSF30 has a disulfide linkage between two cysteine residues in the C-terminal zinc finger motif that is required for the endonuclease activity of the protein, such that treatment with DTT inhibits this activity [57,58]. Exposure of cells to oxidative stress can cause reversible changes in the reactive cysteine residues in many redox-regulated proteins, leading to initiation of cellular signaling events culminating in a stress response [79]. The sensitivity of AtCPSF30 to DTT raises the possibility that this protein is regulated *in vivo* by remodeling of the disulfide bond. Such processes provide an additional conceptual link between AtCPSF30 and cellular signaling.

These considerations may be summarized as follows. Biochemical and molecular studies provide conceptual links between AtCPSF30 and different modes of cellular signaling (namely, calmodulin-mediated and redox-associated). Genetic analyses link AtCPSF30 with a wide range of developmental and physiological responses, and they indicate that only a subset of these involves the interaction of AtCPSF30 with calmodulin (and, thus, with calcium signaling). Biochemical studies indicate that cellular signaling pathways may impact the activities of the protein. Together, they lead to the model shown in Figure 3, in which AtCPSF30 is a conduit that connects cellular signaling with posttranscriptional processes. The nature of these is discussed in the following section.

## 4. The Scope of AtCPSF30-Mediated APA

The roles of AtCPSF30 in so many physiological processes suggest that AtCPSF30-mediated APA is important for the expression of genes associated with these processes. Given the inhibitory effects of calmodulin, and of disulfide bond reduction, on AtCPSF30, it stands to reason that inhibition of AtCPSF30 may be a contributing factor to posttranscriptional controls of gene expression. This suggestion leads to the question—what might be the scope of AtCPSF30-mediated APA? This question has been addressed by global poly(A) site profiling in wild type and *oxt6* mutant *Arabidopsis* plants [68,80]. The results of these studies show that many more than half of all expressed genes have different poly(A) site profiles in the mutant when compared with the wild-type. Moreover, 70% of all of the poly(A) sites seen in the mutant and wild-type (sites those appear only in the wild type or *oxt6* mutant and those appear in both) are present either in the wild type or in the *oxt6* mutant, but not in both backgrounds [80]. These represent AtCPSF30-dependent poly(A) sites, whose usage is determined by the presence or absence of AtCPSF30. These sites are distributed throughout the various genomic locations (3'-UTRs, 5'-UTRs, protein coding regions, and introns). Interestingly, stress-responsive genes are over-represented in the set of genes that possess AtCPSF30-dependent poly(A) sites that lie within 5'-UTRs, protein-coding regions, and introns. Usage of such sites is likely to alter the expression and/or function of the associated gene; thus, this correlation is suggestive of a role for AtCPSF30-mediated APA in the regulation of expression of genes involved in stress responses. This is in accordance with the roles of AtCPSF30 in stress responses that are discussed in the preceding section [69,80].

In animals, APA is typically manifested as the usage of poly(A) sites that lead to short or long mRNA isoforms. Genes subjected to APA in fast growing undifferentiated cells use proximal poly(A) sites and thereby generate shorter mRNA isoforms, which lead to expression at higher levels as they lack possible targets for microRNAs or other regulatory proteins. In contrast, genes subjected to APA in differentiated cells use distal poly(A) sites and generate longer RNA isoforms [3]. While there are many AtCPSF30-dependent sites that fall within 3'-UTRs in *Arabidopsis*, a similar proximal/distal pattern of usage is not seen in the case of AtCPSF30-dependent poly(A) site choice [80]. However, poly(A) sites seen only in the *oxt6* mutant (and thus repressed by AtCPSF30) were found to lack one of the sub-elements (the so-called Near Upstream Element, or NUE), that constitute a canonical plant polyadenylation signal [80,81]. This is reminiscent of so-called proximal sites in animal genes, in that these sites have sub-optimal polyadenylation signals. Thus, while the spatial organization of alternative sites in these two systems may differ, the association of APA with seemingly suboptimal polyadenylation signals may reflect a common mechanism of APA.

The genome-wide studies of poly(A) site choice in the *oxt6* mutant show that AtCPSF30-mediated APA has the potential to affect the expression of a large number of genes in *Arabidopsis*. This is consistent with the numerous phenotypes that are seen in the *oxt6* mutant [68,82]. The genome-wide studies further suggest that cellular signaling pathways have the potential, via inhibition of AtCPSF30, to alter the regulation of a large number of genes at the posttranscriptional level. This is likely to augment or refine other modes of regulation (such as transcriptional control) that are associated with signaling pathways.

## 5. Conclusions and Future Directions

Stress and developmental signals can initiate cellular signaling cascades involving calcium-calmodulin mediated and/or ROS signaling, which can in turn alter the activity of AtCPSF30 and subsequently alter poly(A) site usage in numerous genes. AtCPSF30 plays roles in a number of distinctive developmental processes and physiological responses [68,82]. At the moment, the links between AtCPSF30-mediated APA and downstream effects are not clear. Alterations of the activities of AtCPSF30 (as in cells activated for calcium/calmodulin or redox signaling) may lead to APA of mRNAs encoded by specific downstream genes; the scope of such changes should be large, based on the results of genome-wide poly(A) site profiling in the *oxt6* mutant [80]. The expression of genes encoding these primary targets of AtCPSF30 may thus be altered in diverse ways, as described above. Interestingly, genes that encode putative receptors and receptor-like proteins are among those over-represented in the set of genes subject to AtCPSF30-mediated APA [80]. Thus, AtCPSF30-mediated APA has the potential to impact many gene expression networks via secondary effects involving proteins encoded by the primary targets of AtCPSF30. Additionally, different signaling cascades might alter the activity of AtCPSF30 in different ways. For example, inhibition of RNA binding (such as via interaction with calmodulin) or alteration of the activity of AtCPSF30 via reduction (or oxidation) of the disulfide bond in the third zinc finger motif might affect different mRNAs, thereby leading to APA and subsequent modulation in expression in distinct sets of genes; ultimately culminating into diverse phenotypic responses.

An alternative to this “gene-centric” model may be proposed. The wide-spread APA associated with loss of AtCPSF30 activity (as in the *oxt6* mutant, or perhaps in cells activated for calmodulin signaling or disulfide bond remodeling) has the potential to trigger a significant production of aberrant proteins encoded by mRNAs that are polyadenylated within introns or protein-coding regions. This could in turn activate general protein quality control pathways. Protein quality control has been linked with numerous processes in plants [83,84,85,86], and it is possible that an AtCPSF30-associated induction of these processes may underlie the contributions of AtCPSF30 to different physiological and developmental responses (as depicted in Figure 3). Resolution of this issue (specific *vs*. general modes of action) is an open issue, the answers to which will reveal much about the role of APA in plant growth and development.

Another outstanding question relates to the role of the larger polypeptide encoded by the *AtCPSF30* gene; this polypeptide consists of almost the entire CPSF30 protein, fused to a YTH domain-containing polypeptide. As stated above, the YTH domain binds RNAs possessing N^6^-methyladenosine (m^6^A) [52]. m^6^A-modifications in RNA are ubiquitous, dynamic, and associated with numerous developmental processes in eukaryotes, including plants [87,88,89,90,91,92,93,94,95]. The occurrence of protein modules associated with mRNA 3'-end processing and m^6^A-RNA binding in the same polypeptide is a tantalizing possibility and raises many questions, not the least of which is the possible involvement of this distinctive RNA modification in other aspects of RNA processing (such as polyadenylation).

In terms of APA as a whole, there are several genome-wide studies assessing the impact of several core polyadenylation factors, including CFIm25 (25 kD mammalian cleavage factor 1), CFIm68 (68 kD mammalian cleavage factor 1), PABPN1 (poly(A) binding protein nuclear 1) and CstF64/CstF64τ [96,97,98,99,100,101], on APA in mammals. These studies establish a general paradigm, that alterations in core polyadenylation complex subunits can lead to global changes in poly(A) site choice. The characteristics of the *Arabidopsis oxt6* mutant [80] are consistent with this paradigm, in that the alteration of levels of at least one core plant polyadenylation complex subunit is a means by which posttranscriptional regulation is accomplished. Whether this paradigm holds for other plant polyadenylation factor subunits remains to be determined; however, the answers will help to define the larger scope of APA and its regulation in plants.

## Appendix

**Table A1:** Genbank accessions for CPSF30 orthologs in different species used for the phylogenetic analysis.

Species	Genbank accessions used for the phylogeny for small polypeptide	Genbank accessions used for the phylogeny for large polypeptide
*Camelina sativa*	XP_010460838	XP_010478453
*Brassica napus*	CDX90251, CDX98205, CDX77591	CDX77591, CDX98205
*Brassica rapa*	XP_009102697, XP_009109585	XP_009109585, XP_009102697
*Populus trichocarpa*	XP_006377637	XP_002300333
*Arabidopsis lyrata*	XP_002893618	XP_002893618
*Capsella rubella*	XP_006306994	XP_006306994
*Cucumis sativus*	XP_004156192, XP_004141524	XP_004141524
*Cucumis melo*	XP_008459517, XP_008445183	XP_008459517, XP_008445183
*Citrus sinensis*	XP_006468290	XP_006468290
*Vitis vinifera*	XP_002281594	XP_002281594
*Malus domestica*	NP_001280880, XP_008372260	NP_001280880
*Theobroma cacao*	XP_007041140	XP_007041140
*Medicago trancatula*	KEH37048	KEH37048
*Cicer arietinum*	XP_004486563	XP_004486563
*Beta vulgaris subsp vulgaris*	XP_010687042	XP_010687042
*Solanum lycopersicum*	XP_004231555, XP_004233145	XP_004231555, XP_004233145
*Solanum tuberosum*	XP_006359103, XP_006352991	XP_006359103, XP_006352991
*Trifolium pratense*	Predicted from RNAseq	Predicted from RNAseq
*Fragaria vesca subsp vesca*	XP_004295608	XP_004295608
*Hordeum vulgare subsp vulgare*	BAJ96745	BAJ96745
*Sorghum bicolor*	XP_002437445	XP_002437445
*Oryza sativa japonica*	NP_001058359	NP_001058359
*Brachypodium distachyon*	XP_003563404	XP_003563404
*Phaseolus vulgaris*	XP_007147504	XP_007147504
*Glycine max*	XP_003546247, XP_003534764	XP_003546247, XP_003534764
*Jatropha curcas*	KDP34942	KDP34942
*Ricinus communis*	XP_002523201	XP_002523201
*Prunus persica*	XP_007214175	XP_007214175
*Eucalyptus grandis*	XP_010056977	XP_010056977
*Setaria italica*	XP_004966206	XP_004966206
*Sesamum indicum*	XP_011085214	XP_011085214
*Aegilops tauschii*	EMT09537	EMT09537
*Physcomitrella patens*	XP_001753463	XP_001753463
*Chlamydomonas reinhardtii*	g18261.t1 (from Phytozome)	
*Volvox carteri*	XP_002947795	
*Micromonas pusilla*	XP_003056614	
*Saccharomyces cerevisiae*_EC1118	CAY87059	
